# MiR-448 promotes glycolytic metabolism of gastric cancer by downregulating KDM2B

**DOI:** 10.18632/oncotarget.8020

**Published:** 2016-03-10

**Authors:** Xuehui Hong, Yang Xu, Xingfeng Qiu, Yuekun Zhu, Xing Feng, Zhijie Ding, Shifeng Zhang, Lifeng Zhong, Yifan Zhuang, Chen Su, Xinya Hong, Jianchun Cai

**Affiliations:** ^1^ Department of Gastrointestinal Surgery, Zhongshan Hospital of Xiamen University, Xiamen, Fujian, China; ^2^ Institute of Gastrointestinal Oncology, Medical College of Xiamen University, Xiamen, Fujian, China; ^3^ Department of General Surgery, The Second Affiliated Hospital of Harbin Medical University, Harbin, Heilongjiang, China; ^4^ Department of General Surgery, The First Affiliated Hospital of Harbin Medical University, Harbin, China; ^5^ Department of Radiation Oncology, Cancer Institute of New Jersey, Rutgers University, New Brunswick, New Jersey, USA; ^6^ Department of Medical Imaging and Ultrasound, Zhongshan Hospital of Xiamen University, Xiamen, Fujian, China

**Keywords:** miR-448, glucose metabolism, gastric cancer, lysine (K)-specific demethylase 2B, mitochondria respiration

## Abstract

MicroRNAs are critical in various human cancers, including gastric cancer (GC). However, the mechanism underlying the GC development remains elusive. In this study, we demonstrate that miR-448 is increased in GC samples and cell lines. Overexpression of miR-448 facilitated the proliferation of GC cells by stimulating glycolysis. Mechanistically, we identified KDM2B, a reader for methylated CpGs, as the target of miR-448 that represses glycolysis and promotes oxidative phosphorylation. Overexpression of miR-448 reduced both the mRNA and protein levels of KDM2B, whereas KDM2B re-expression abrogated the miR-448-mediated glycolytic activities. Furthermore, we discovered Myc as a key target of KDM2B that controls metabolic switch in GC. Importantly, a cohort of 81 GC tissues revealed that miR-448 level closely associated with a battery of glycolytic genes, in which KDM2B showed the strongest anti-correlation coefficient. In addition, enhanced miR-448 level was significantly associated with poor clinical outcomes of GC patients. Hence, we identified a previously unappreciated mechanism by which miR-448 orchestrate epigenetic, transcriptional and metabolic networks to promote GC progression, suggesting the possibility of therapeutic intervention against cancer metabolic pathways.

## INTRODUCTION

Warburg effect or aerobic glycolysis is a common feature of cancer cells. Otto Warburg firstly observed and reported that contrary to normal differentiated cells that use mitochondrial oxidative phosphorylation (OXPHOS) as a main source for energy production, tumor cells are addictively dependent on glycolysis and display high glycolytic rate with production of lactate even in an oxygen-rich condition [1]. This shift in metabolism is believed to provide metabolic needs for the rapid proliferating cancer cells to grow, rather than energy production [2]. The understanding of the control of this metabolic shift is pivotal to identify potential targets for cancer therapeutics.

Gastric cancer (GC) is the second most common cause of cancer-related mortality [3]. Although accumulating evidence shows that various genetic alterations and a combination of environmental factors cause tumorigenesis and progression of GC [4], the molecular mechanisms underlying the pathogenesis of GC remain to be fully defined. Given that the prognosis is still poor, with a long-term survival rate of only 30–40% for patients under 60 years old and 10% for patients above 60 years old [5], understanding the fundamental mechanisms of GC and developing better treatment methods remain challenging.

MicroRNAs (miRNAs) are a class of non-coding, 20–25 nucleotide-long RNAs. Through imperfect pairing with the 3′-untranslated regions (3′-UTRs) of mRNA, they can regulate target gene expression at the post-transcriptional level by inhibiting translation or by increasing messenger RNA (mRNA) degradation [6]. Recent evidence indicates that miRNA levels are closely correlated with tumor metastasis and proliferation [7]. For example, miR-145 and miR-218 suppress GC cell metastasis by inhibiting the expression of N-cadherin and Robo1, respectively [8]. And research on miRNAs and their molecular mechanisms in GC have opened a new door for therapeutic strategies.

The present study is to identify and characterize the miR-448-targeted metabolic genes with an attempt to evaluate the potential of reversing aerobic glycolysis in GC.

## RESULTS

### MiR-448 is overexpressed in GC and associated with poor survival

**Figure 1:**
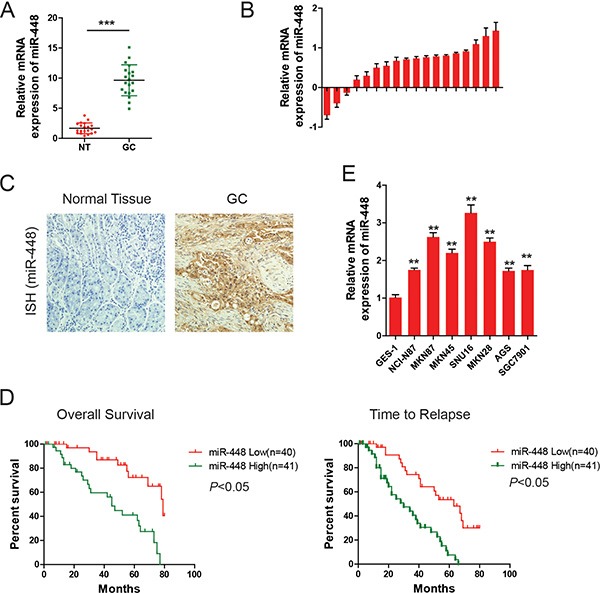
MiR-448 is increased in GC and associated with poor prognosis (**A**) Scatter dot plots show relative mRNA levels of miR-448 in GC and adjacent non-tumor (NT) samples using data from The Cancer Genome Atlas. (**B**) Forest plots show miR-448 expression in GC tumor and adjacent non-tumor tissues. (**C**) Representative images of miR-448 expression by ISH. (**D**) Kaplan-Meier curves of overall survival (OS) and time to relapse (TTR) for GC patients with high/low miR-448 expression. (**E**) Relative expression of miR-448 in GC cell lines.

We firstly investigated the miR-448 expression pattern in GC. The results indicated that the expression level of miR-448 was found to be markedly higher in GC samples than in adjacent non-tumor tissues (Figure 1A), which was further confirmed using 18 paired GC samples and non-tumor tissues (Figure 1B). Moreover, *in situ* hybridization (ISH) indicated that the expression of miR-448 were significantly upregulated in 17 of 20 patients compared with the adjacent non-tumor samples (Figure 1C). Kaplan–Meier analysis showed that the survival time (overall survival) and time to relapse (TTR) of patients with low miR-448 expression were significantly longer compared with high miR-448 expression (Figure 1D). Additionally, high miR-448 expression was correlated with poor histological differentiation, tumor size and distant metastasis (Supplementary Table 1). miR-448 expression in different human cell lines was also investigated using RT-PCR. The expression of miR-448 in the GC cell lines, NCI-N87, MKN87, SNU16, SGC7901, AGS, MKN28, and MKN45 was significantly higher than that in the normal gastric mucous cell line GES-1 (Figure 1E). This is consistent with our clinical findings that human GC tissues have high miR-448 expression.

### miR-448 promotes GC growth

**Figure 2:**
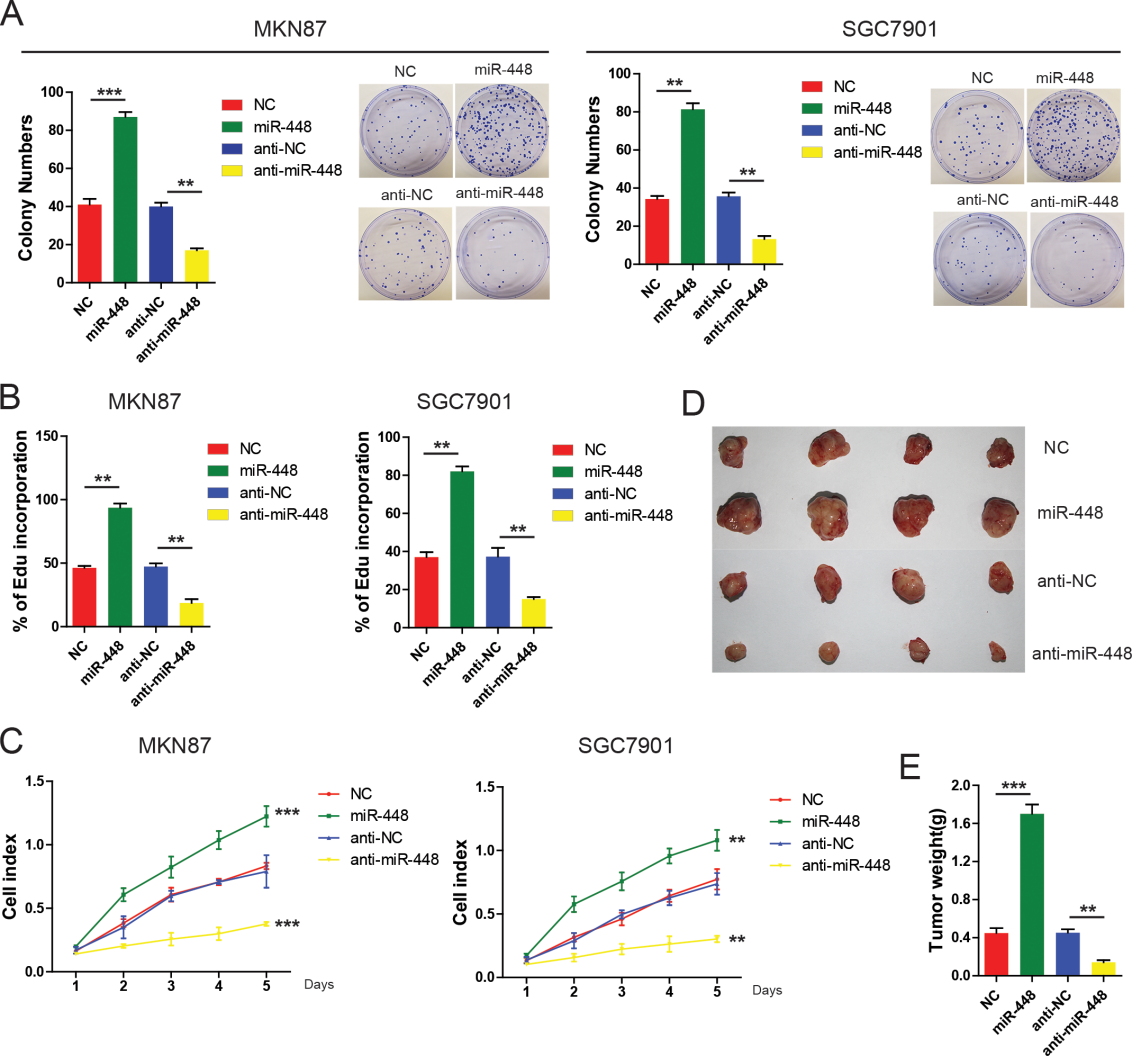
miR-448 promotes GC cell growth and carcinogenicity *in vitro* and *in vivo* Colony formation assays (**A**), DNA synthesis (**B**), and real-time cell proliferation assays (**C**) in GC cells with miR-448 overexpression or inhibition. (**D**) Xenograft tumors in nude mice. (**E**) Overexpression of miR-448 increases weight of the xenograft tumors.

To investigate the role of miR-448 in GC, the colony formation assay was performed. It was found that miR-448 mimics increased colony-forming ability of GC cells (Figure 2A). Both 5-ethynyl-2′-deoxyuridine (Edu) incorporation and real-time cell proliferation assays also indicated that exogenous miR-448 transfection increased growth of GC cells (Figure 2B and 2C). Conversely, compared with control cells, treatment with an miR-448 inhibitor impaired cell growth (Figure 2A, 2B and 2C). Consistent with these findings *in vitro*, miR-448 remarkably enhanced the tumorigenic ability of GC cells *in vivo* (Figure 2D). Compared with the control group, tumor weight was dramatically decreased in the anti-miR-448 group (Figure 2E). Together, our data suggest that miR-448 promotes proliferation in GC.

### miR-448 promotes glycolysis in GC

**Figure 3:**
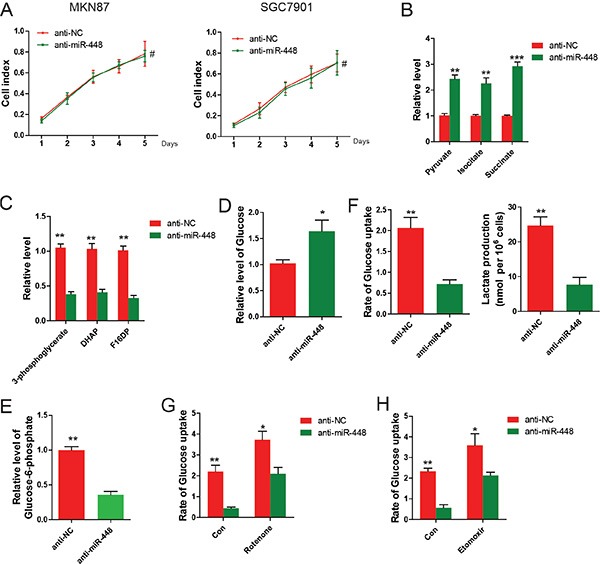
miR-448 knockdown decreases glycolytic and increases mitochondrial oxidative metabolism (**A**) Growth curves of anti-NC and anti-miR-448 GC cells cultured in medium containing galactose. (**B**) Measurement of TCA cycle intermediates and glycolytic intermediates (**C**) in anti-NC and anti-miR-448 GC cells. Measurement of glucose (**D**) and glucose-1-phosphate (**E**) in anti-NC and anti-miR-448 GC cells. (**F**) Measurement of glucose uptake and lactate production in anti-NC and anti-miR-448 GC cells. (**G**) Measurement of glucose uptake in anti-NC and anti-miR-448 GC cells treated with or without 100 nM rotenone for 24 hr before measuring glucose uptake. (**H**) Measurement of Glucose uptake in anti-NC and anti-miR-448 GC cells treated with or without 50 μg/ml etomoxir for 24 hr before measuring glucose uptake.

To investigate whether aerobic metabolism of glucose was required by this enhanced GC growth, GC cells were cultured in medium containing galactose instead of glucose, which inhibits glycolytic flux and forces the cell to rely on mitochondrial oxidative phosphorylation [9]. It was found that GC cells treated with anti-NC and anti-miR-448 repspectively proliferated at the similar rate, suggesting that the increased glycolysis was necessary for proliferation of anti-miR-448 GC cells (Figure 3A). Similarly, GC cells transfected with NC or miR-418 showed no difference at proliferation rate under glucose deprivation.(data not shown). Therefore, it is possible that miR-448 promotes GC growth by controlling the balance between mitochondrial oxidative and glycolytic metabolism. To test this idea, we first measured metabolites from anti-miR-448 GC cells. It was found that TCA cycle metabolites were increased, whereas intermediates of glycolysis were decreased (Figure 3B and 3C). Conversely, the TCA cycle metabolites were decreased while intermediates of glycolysis were upregulated in GC cells with miR-448 overexpression (data not shown). Moreover, compared with the control group, levels of intracellular glucose from anti-miR-448 cells were higher (Figure 3D), whereas levels of glucose-1-phosphate, a product of glycogenolysis, were decreased (Figure 3E). Constantly, compared with the control cells, anti-miR-448 cells consumed less glucose and extruded less lactate into the medium (Figure 3F). These data suggest that miR-448 redirects GC cellular metabolism and promotes glycolysis.

To investigate whether miR-448 promotes glycolysis as part of a compensatory response due to decreased oxidative capacity, glucose uptake were measured using anti-448 GC cells in the presence of rotenonea (a mitochondrial respiratory inhibitor) or etomoxir (an inhibitor of mitochondrial fatty acid oxidation). We found that glucose uptake was decreased in the presence of both rotenone (Figure 3G) and etomoxir (Figure 3H), suggesting that diminished glycolysis in anti-miR-448 cells does not result solely from nonspecific compensation due to increased mitochondrial oxidative functions.

### miR-448 stimulates glycolysis by repressing KDM2B

Given that miRNAs have been reported to play critical roles through repressing their target genes, thus it is impossible that miR-448 directly upregulates glycolysis-related genes. miR-448 might inhibit a transcriptional repressor which promoted oxidative phosphorylation and decreased expression of glycolysis-related genes. To test this idea, using a bioinformatic method, we screened transcriptional repressors that have predicted miR-448 binding sites in their 3′ UTRs, and are upregulated in Dicer −/− *versus +/+* GC cells, but downregulated upon miR-448 transfection. KDM2B was considered as a candidate of transcriptional repressors because it had recently shown promoting OXPHOS [10].

**Figure 4:**
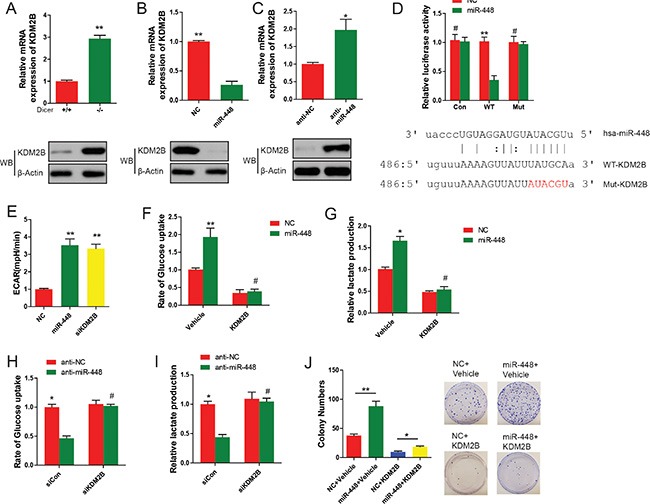
KDM2B is a direct target of miR-448 and its suppression promotes glycolytic metabolism in GC cells (**A**) Analysis of KDM2B expression in WT and Dicer−/− and +/+ MEFs using qRT–PCR and Western blotting. (**B**) Analysis of KDM2B expression in GC cells transfected with miR-448 mimic using qRT–PCR and Western blotting. (**C**) Analysis of KDM2B expression in anti-NC and anti-miR-448 GC cells using qRT–PCR and Western blotting. (**D**) Luciferase reporter assay for KDM2B WT and Mut 3′ UT Rs. (**E**) ECAR assays. (**F**) Cellular glucose uptake and lactate production were measured (**G**) in GC cells transfected with or without miR-448 or KDM2B. (**H**) and (**I**) Cellular glucose uptake and lactate production were measured in in GC cells transfected with or without anti-miR-448 or KDM2B siRNA. J. KDM2B was introduced with or without miR-448, and positive colonies were counted on day 18 after virus infection.

Our results indicated that upon loss of miRNAs via Dicer knockout, the higher expression levels of both KDM2B mRNA and protein in MEFs were observed (Figure 4A). Consistent with these findings, miR-448 significantly decreased the expression of KDM2B at both mRNA and protein levels (Figure 4B and 4C). To examine whether miR-448 directly inhibits KDM2B, two luciferase reporters by inserting WT or mutated (Mut) 3′ UTR region of KDM2B gene containing the potential miR-448 binding site into psiCheck2 vector were generated (Figure 4D). It was found that the transfection of miR-448 significantly decreased the luciferase activity of the reporter containing WT but not Mut 3′ UTR. These results demonstrated that KDM2B is a direct target of miR-448 in GC cells.

Extracellular acidification rate (ECAR) index is the surrogate of glycolysis level. To tested whether KDM2B is a functional target of miR-448 in promoting glycolysis. The effect of KDM2B knockdown and miR-448 overexpression on extracellular acidification rate (ECAR) index in GC cells was compared. No discernible difference was observed (Figure 4E). Overexpression of KDM2B in GC cells also reverted enhanced cellular glucose uptake and lactate production in miR-448 overexpressed cells (Figure 4F and 4G). Conversely, siRNA against KDM2B reverted the reduced glucose uptake and lactate production in anti-miR-448 cells (Figure 4H and 4I). More importantly, overexpression of exogenous KDM2B reverted the increased GC proliferation induced by miR-448 (Figure 4J). Together, our data suggest that KDM2B is a direct target gene of miR-448, and its inhibition is critical and essential for miR-448-promoted glycolysis.

### KDM2B suppresses glycolysis by inhibiting the transcription of Myc

Next, we wondered to know how KDM2B inhibits glycolysis in GC cells. A plausible explanation for how KDM2B inhibits glycolysis is that KDM2B directly represses the transcription of critical glycolytic genes by binding to their promoters. Transcription factor Myc was focused on because it is well known to promote glycolysis in numerous cancer cells [11]. We hypothesized that Myc might function as a downstream target of KDM2B.

**Figure 5:**
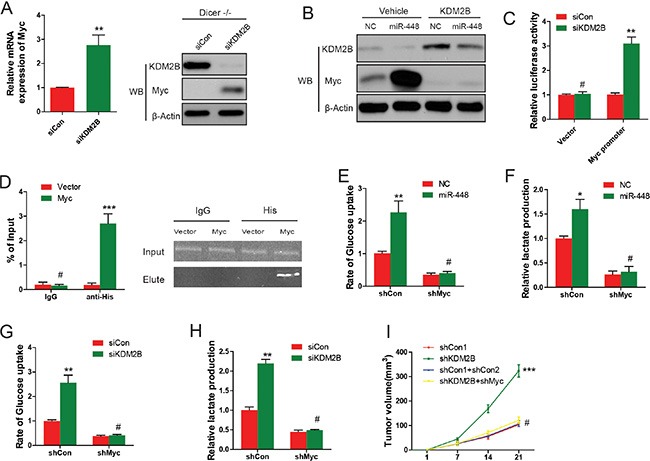
miR-448-mediated inhibition of KDM2B induces Myc expression and glycolysis (**A**) Myc expression in GC cells or Dicer−/− MEF cells infected with KDM2B siRNAs was analyzed using qRT–PCR and Western blotting. (**B**) Myc protein expression in GC cells stably expressing KDM2B and further infected with miR-448 was analyzed using Western blotting. (**C**) The activity assay of Myc promoter upon KDM2B depletion by siRNAs in GC cells. Empty pGL3-enhancer plasmid was used as a control. (**D**) ChIP–qPCR analysis of Myc promoter. (**E**) and (**F**) Glucose uptake and lactate production were measured in GC cells expressing Myc shRNA and further infected with miR-448. (**G**) and (**H**) Glucose uptake and lactate production were measured in GC cells stably expressing Myc shRNA and further infected with KDM2B siRNA. (**I**) shMyc significantly reverted the positive effect shKDM2B on GC growth.

It was found that KDM2B knockdown significantly increased Myc mRNA and protein level expression in GC cells (Figure 5A). Moreover, overexpression of exogenous KDM2B reverted the increase in Myc induced by miR-448 (Figure 5B), suggesting that miR-448 upregulated Myc expression through inhibiting KDM2B. To investigate whether KDM2B directly inhibited the transcription of Myc, promoter activity assay was performed. It was found that siRNA against KDM2B greatly enhanced the activity of a luciferase reporter bearing Myc promoter (Figure 5C). ChIP analysis showed that KDM2B was markedly recruited to the promoter region of Myc (Figure 5D). Together, these results showed that KDM2B directly binds to Myc promoter to inhibit Myc expression, and that its inhibiton by miR-448 is necessary for maintaining the high expression level of Myc in GC cells.

The role of Myc in metabolic regulation in GC cells was then investigated through Myc loss or gain of function experiments, respectively. It was found that overexpression of Myc, similar to overexpression of miR-448, promoted glucose uptake and lactate production (Figure 5E and 5F). To further confirm the role of Myc in miR-448/KDM2B pathway, Myc firstly was knocked down and then miR-448 or KDM2B siRNA was introduced into in GC cells. The promoting effects on glucose uptake and lactate production by miR-448 or siKDM2B were completely abolished upon knocking down Myc (Figure 5G and 5H), suggesting that Myc is critical for miR-448/KDM2B-regulated glycolysis in GC cells.

To investigate the rescue function of Myc, we introduced shMyc into GC cells expressing shKDM2B. As downstream factors regulated by KDM2B, shMyc significantly reverted the positive effect shKDM2B has on GC growth (Figure 5I).

### Clinical relevance of miR-448, KDM2B and Myc expression in patients with GC

**Figure 6:**
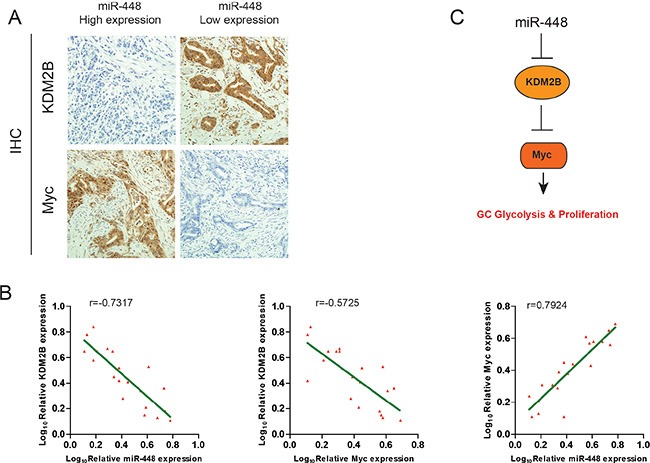
Clinical relevance of miR-448, KDM2B and Myc Expression in patients with GC (**A**) Representative images of IHC staining for KDM2B and Myc in low and high miR-448 expression GC cases. Bars: 50 μm. (**B**) Correlation between miR-448 expression and KDM2B and Myc expression in the clinical samples. (**C**) Schematic diagram of the regulatory pathway from miR-448 to glycolysis.

Given that miR-448 downregulates KDM2B and upregulates Myc expression in GC cells, we evaluated whether this pathway also functions *in vivo*. Therefore, miR-448 expression was investigated using *in situ* hybridization (ISH) in a cohort of 60 GC samples, followed by immunohistochemistry (IHC) staining for KDM2B and Myc. As shown in Figure 6A, GC with high miR-448 expression exhibited a more malignant phenotype, whereas GC with low miR-448 expression exhibited more epithelial-like characteristics with the formation of gland-like tubular structures. Consistently, we observed an inverse expression pattern between KDM2B and miR-448 or Myc, whereas a positive expression pattern between miR-448 and myc was observed (Figure 6B). These clinical data further support mechanism postulating that miR-448 suppresses the expression of KDM2B that directly inhibit Myc to maintain increased glycolysis in GC cells (Figure 6C).

## DISCUSSION

In this study, we demonstrated that expression of miR-448 is significantly elevated in GC, and that it increases the expression of Myc, the rate-limiting enzyme for glycolysis, by directly inhibiting KDM2B. Thus, our study provides evidence that miR-448 is a novel positive regulator of glycolysis and a potential therapeutic target for treatment of patients with GC.

Despite the general increase of “Warburg effect” requirements in many cancers, several recent studies have revealed unanticipated tumor metabolism requirements for OXPHOS. Thus, the bio energetic type of tumors can change widely from glycolysis to OXPHOS [9]. Several genes were shown to contribute to tumor growth by enhancing OXPHOS. For example, KDM2B has recently been shown being involved in tumor metabolism. But unexpectedly, KDM2B promoted *K-RAS*–mutated pancreatic cancer through upregulating a module of metabolic genes involved in OXPHOS in a mouse allograft model [10]. It was also reported that KDM2B, as a bona fide oncogene, directly binds to and activates OXPHOS genes, not glycolytic-related genes, and subsequently contributes to the development of leukemia involving metabolic proliferative advantage [11]. On the contrary, our findings indicate that KDM2B functions as a GC suppressor by inhibiting Myc-mediated glycolysis. Therefore, our data may enrich the mechanism of the KDM2B's regulatory role in various cancers. However, a number of questions remain to be answered, including possible involvement of other factors. The search for other factors will no doubt contribute to elucidating the mechanism by which dysregulation of KDM2B leads to GC.

Because each miRNA is estimated to target several hundred distinct genes, their roles are conceivably as important as TFs or signaling molecules in controlling various cellular processes [12]. The functional characterization of miRNA heavily relies on the identification of its targets and its effects on their expression. miR-448 has been characterized as a tumor suppressor in breast cancer, ovarian cancer and hepatocellular carcinoma [13–15]. A series of molecules have been reported as the direct targets of miR-448 in different cell contexts, such as CXCL12, ROCK2, and special AT-rich sequence-binding protein-1 (13, 14, 15). However, the opposite effect has also been reported. For example, miR-448 might promote non-small cell lung cancer (NSCLC) progression [16], which is consistent with our data. And we identified KDM2B as a new target gene of miR-448. These conflicting findings imply that the ambiguous role of miR-448 in different tumor types requires further exploration.

Myc and HIF1A are the best-known transcriptional regulators controlling expression of glycolysis genes, such as *GLUT1*, *HK2*, *PDK1*, and *LDHA*, whose expression levels are highly elevated in cancer cells [17, 18]. Recent studies have suggested that c-Myc has been associated with the production of many non-coding RNAs, including miRNAs [19]. miR-448 has been mapped to the fourth intron of *HRT2C* on chromosome X [13]. Using MatInspector software, an algorithmic prediction of potential transcription factor binding sites identified one putative-binding site (E-box: CACGTG) for Myc within the region of miR-448 promoter, suggesting that miR-448 may be a downstream factor of Myc. However, it remains to confirm if Myc is indeed recruited to the miR-448/HRT2C promoter region to promote miR-448 expression, thus establishing one feedback loop in GC cells.

In conclusion, we found that novel roles of miR-448 are linked to glycolysis by KDM2B/Myc *axis* in GC, suggesting that miR-448 might be a prognostic biomarker and potential target for future GC treatment.

## MATERIALS AND METHODS

### Cell lines and cell cultures

Human GC cell lines (AGS, MKN28, MKN87, NCI-N87, SNU16, SGC7901, and MKN45) and human gastric mucosa epithelial cells (GES-1) were purchased from the Cell Resource Center, Shanghai Institute of Biochemistry and Cell Bank at the Chinese Academy of Sciences, propagated in RPMI1640 (Invitrogen) supplemented with 10% fetal bovine serum [FBS (HyClone)] under a humidified air atmosphere containing 5% carbon dioxide. HEK-293T cells were cultured in DMEM (Gibco-BRL) with 10% FBS.

### RNA extraction and quantitative qRT-PCR

Total RNA from tissues and cells were extracted using the TRIZOL reagent (Invitrogen) and reverse transcribed by using the M-MLV RT kit (Promega). For the detection of mature miR-448, isolation of total RNA from cells was performed using the miRVana miRNA Isolation Kit (Ambion, USA). According to the manufacturer's instructions, miR-448 was investigated using the miRVana real-time PCR miRNA Detection Kit and real-time PCR Primer Sets. The primers for real-time PCR were shown as below. miR-448: Forward primer: TTATTGCGATGTGTTCCTTATG, Reverse primer: ATGCATGCCACGGGCATATACACT. U6 small nuclear RNA was used for normalization. Real-time PCR assay was performed on ABI PRISM7500 system (Applied Biosystems, USA).

### Plasmid construction and stable/transient transfection of anti-miR-448

For the transient inhibition of miR-448, 50 nM miR-448 inhibitor oligonucleotide (Ambion) were transfected into indicated cells using Lipofectamine 2000 (Invitrogen) according to the manufacturer's instructions. To generate stable miR-448-depleted GC cells transfectants, anti-miR-448 sequences were amplified from miRZip-448 construct (System Biosciences) and subcloned into pSilencer4.1(Ambion) polyclone sites with *Hin*dIII and *Bam*HI sites. GC cells were then transfected with the pSILENCER Expression Vector containing the antisense sequence of miR-448. Cells were selected with puromycin 48 h after transfection and then diluted to perform clonal selection.

### Luciferase reporter assay

HEK293 cells were seeded in 96-well plates. KDM2B 3′-UTR, mutated KDM2B 3′-UTR, or control luciferase reporter plasmid (GeneCopoeia) was co-transfected with either mirVana^™^ miR mimic hsa-miR-448 or mirVanaTM miR mimic negative control (NC; Ambion) using FugeneHD:DNA ratio of 3:1 (Promega), or Lipofectamine 3000 (Thermofisher, USA). Luciferase activity was measured with Secre-Pair^™^ Dual-Luciferase Reporter Assay (GeneCopoeia).

### Clinical sample preparation

All 81 patients with GC were selected after gastric resection and pathological confirmation in the First Affiliated Hospital of Harbin Medical University, including males and females aged 60.5 ± 8.1 years who had not received preoperative radiotherapy or chemotherapy. Cancer and para-carcinoma tissues (approximately 1.0 cm × 0.5 cm × 0.5 cm) were taken from every patient, and the specimens were rapidly frozen in liquid nitrogen and subsequently transferred to −80°C conservation. This study was performed in accordance with the ethical standards of the Declaration of Helsinki. All participants provided their written informed consent to participate in this study, and the ethics committee approved this consent procedure.

### Chromatin immunoprecipitation (ChIP) assay

Briefly, Dicer−/− MEF cells overexpressing His-KDM2B or vector with different treatment were fixed with 1% formaldehyde for 15 min at room temperature, and terminated by a final concentration of 0.125 M glycine. Then cells were lysed using 300 μl lysis buffer (50 mM Tris-HCl, pH 8.0, 150 mM NaCl, 5 mM EDTA, 1% Nonidet P-40, 0.5% deoxycholate, and protease inhibitors). The cell lysates were sonicated in ice water bath to yield chromatin fragments about 600 bp, as assessed by agarose gel electrophoresis. After centrifugation at 13,000 rpm for 10 min, the supernatants were taken and pre-cleared for 15 min at 4°C via incubation with 30 μl of protein A-Sepharose beads and sheared salmon sperm DNA. After centrifugation at 13,000 rpm for 5 min, the supernatants were divided into three equal parts: one for input, the other two for immunoprecipitation with or without His antibody or IgG. The next day, the immune complexes were precipitated with protein A-Sepharose beads and sheared salmon sperm DNA, then the beads were collected after washed twice with the wash buffer I (20 mM Tris-HCl, pH 8.1, 150 mM NaCl, 0.1% SDS, 1% Triton X-100, and 2 mM EDTA), followed by wash buffer II (20 mM Tris-HCl, pH 8.1, 500 mM NaCl, 0.1% SDS, 1% Triton X-100, and 2 mM EDTA), and wash buffer III (10 mM Tris-HCl, pH 8.1, 0.25 M LiCl, 1% Nonidet P-40, 1% deoxycholate, and 1 mM EDTA), and the final wash buffer IV (10 mM Tris-HCl, pH 8.1, and 1 mM EDTA). The immunoprecipitates were eluted by 200 μl elution buffer (1% SDS and 0.1 M NaHCO_3_), followed by incubation at 65°C overnight. The next day, DNA of each sample was isolated, and PCR was performed to amplify the Myc promoter segments containing a KDM2B binding site. The primers used were as follows: forward, 5′-GGCCCGTCGTTTCGCCATC-3′; reverse, 5′ -CAAGCTCTTCGGTGTGGT-3′. The PCR products were subjected to 2% agarose gel electrophoresis.

### Western blot analyses

Cells were lysed with RIPA buffer (BIO-RAD). Lysates were separated by SDS polyacrylamide gel electrophoresis (SDS-PAGE), followed by transferring to polyvinylidene difluoride (PVDF) membranes (Millipore, USA). After blocking with 5% non-fat dry milk at room temperature for 1 h, the membranes were incubated overnight at 4°C with primary antibodies. After washing with TBST, the membranes were incubated with secondary antibodies (BIO-RAD) at room temperature for 1 hour. Protein bands were visualized with the electrochemiluminescence (ECL) detection system, and the expression levels of the proteins were evaluated using Image-Pro Plus 6.0 software (Media Cybernetics, USA).

### Cell proliferation and colony formation assays

The real-time cell proliferation assays were performed using an xCELLigence RTCA DP (Roche) instrument following the manufacturer's instructions, and changes in cell numbers were reflected by concomitant changes in the Cell Index. For the colony formation assay, 1 × 10^3^ cells were plated in each well of a 6-well plate and incubated at 37°C for 1–2 weeks. The cells were fixed with 4% paraformaldehyde and stained with 1% crystal violet (Sigma-Aldrich). Megascopic cell colonies were counted and analyzed.

### Cellular glucose-6-phosphate assays

The cellular levels of glucose-6-phosphate was measured using a Glucose-6-phosphate Fluorometric Assay kit (Cayman, USA). All values were normalized to total protein levels.

### 5-Ethynyl-2′-deoxyuridine (EdU) incorporation assay

Cells were incubated with EdU (final concentration, 10 μM) for 1.5 h and analyzed using a Click-iT^®^ EdU Alexa Fluor^®^ Imaging Kit (Molecular Probes, USA) according to the manufacturer's instructions. The number of EdU-positive cells was counted.

### Measurement of cellular respiration and the extracellular acidification rate (ECAR)

The mitochondrial oxygen consumption rate (OCR) and ECAR were measured using a Seahorse Bioscience XF24 extracellular flux analyzer (Seahorse Bioscience). Before the day of the assay, the cartridge sensor was hydrated overnight with 1 mL Seahorse Bioscience XF24 Calibration Buffer at 37°C without CO2. Indicated cells were seeded in an XF24 Islet Capture Microplate and the growth medium was replaced with serum-free DMEM/F12 lacking sodium bicarbonate. Cells were then incubated at 37°C in a non-CO2 incubator for 1 h. OCR and ECAR values were monitored under basal condition and measured after the injection of oligomycin (1 μM), FCCP (carbonyl cyanide p-trifluoromethoxyphenylhydrazone, 1 μM), and antimycin A (1 μM) to the well in succession. OCR and ECAR results were analyzed using the Seahorse XF-24 software. Every point represents an average of five different wells.

### Glucose consumption and lactate production

Glucose consumption and lactate production were measured from supernatants of the cultured cells. Metabolite concentrations were quantified on deproteinized samples using specific enzymatic assays on a CMA600 analyzer (CMA Microdialysis AB, Sweden). Glucose consumption and lactate production were normalized to protein content using the Pierce BCA Protein assay (Thermo Scientific). To efficiently detect differences in metabolite concentrations between SiHa WT and SiHa ρ0 supernatants, a low glucose (1 g/L) medium was used.

### Xenograft mice tumor model

Four-week-old Balb/c nude mice were purchased from the Shanghai SLAC Laboratory Animal Center (Shanghai, China) and maintained under specific pathogen-free conditions. All experimental procedures involving animals were performed in accordance with the Guide for the Care and Use of Laboratory Animals and were approved by the Animal Experimental Ethics Committee of Harbin Medical University. Indicated cells were collected by centrifugation, and suspended in culture medium. A 150 μL sample of culture medium containing 1 × 10^7^ cells was injected subcutaneously into the dorsal flank of each nude mouse. The mice were monitored every week for the growth of tumors, and euthanized after 5 weeks. The tumor xenografts were dissected and weighed after the deaths of the mice.

### Statistical analysis

The data were presented as mean ± standard deviation, with at least three replicates used for each data point. Unless otherwise indicated, a paired Student's *t* test or one-way analysis of variance (ANOVA). Duncan's multiple range test was performed for each experimental group to assess the statistical significance against respective controls. *P < 0.05, **P < 0.01, ***P < 0.001, ^#^*P* > 0.05.

## SUPPLEMENTARY MATERIAL TABLES



## Abbreviations

OXPHOS: Oxidative Phosphorylation
GC: Gastric cancer
KDM2B: Lysine (K)-specific Demethylase 2B
OCR: Oxygen Consumption Rate
ECAR: Extracellular Acidification Rate
Edu: 5-ethynyl-2′-deoxyuridine
