# Supplementary material for: MiR-448 promotes glycolytic metabolism of gastric cancer by downregulating KDM2B
Source: Oncotarget. 2016 Mar 10;7(16):22092–102. doi: 10.18632/oncotarget.8020 (PMC5008346; doi:10.18632/oncotarget.8020)
Supplement: Supplementary file 1 [file oncotarget-07-22092-s001.pdf]

MiR-448 promotes glycolytic metabolism of gastric cancer by downregulating KDM2B

Supplementary Materials

Supplementary Table S1: Relationship between miR-448 Expression and Clinicopathologic Features of GC Patients (*n* = 81)

Features		Relative miR-448 expression		*P* Value
		Low (*n* = 40)	High (*n* = 41)	
Gender	Male	29	31	NS
	Female	11	10	
Age	≤ 50	21	23	NS
	> 50	19	18	
Histological Differentiation	Poor	6	21	< 0.01
	Moderate	11	16	
	Well	23	4	
Tumor size (cm)	≤ 5	27	11	< 0.01
	> 5	13	30	
TNM Stage	I–II	25	28	NS
	III–IV	15	13	
LN metastasis	Yes	6	10	NS
	No	34	31	
Distant metastasis	Yes	4	16	< 0.01
	No	36	25	

Note: GC patients were divided into miR-448 'High' group (Relative fold change was higher than the median) and 'Low' group (Relative fold change was lower than the median).

Abbreviations: LN, lymph node; TNM, tumor-nodes-metastasis; NS, not significant between any groups. Differences among variables were assessed by χ^2 or Fisher's exact χ^2 test.
